# Supplementary material for: Sea Lamprey Alarm Cue Comprises Water- and Chloroform- Soluble Components
Source: J Chem Ecol. 2022 Oct 13;48(9-10):704–17. doi: 10.1007/s10886-022-01384-0 (PMC9559537; doi:10.1007/s10886-022-01384-0)
Supplement: Supplementary file 1 — Supplementary Material 1 [file 10886_2022_1384_MOESM1_ESM.docx]

Journal of Chemical Ecology

Research article

SEA LAMPREY ALARM CUE COMPRISES WATER- AND CHLOROFORM- SOLUBLE COMPONENTS

EMILY L. MENSCH*^1^, AMILA A. DISSANAYAKE^2^, MURALEEDHARAN G. NAIR^2^, and C. MICHAEL WAGNER^1^

*^1^Department of Fisheries and Wildlife, Michigan State University, East Lansing, MI 48824*

*^2^Department of Horticulture, Michigan State University, East Lansing MI 48824*

**Corresponding Author**

Emily Mensch: [Emily.mensch@gmail.com](mailto:Emily.mensch@gmail.com), ORCID 0000-0003-3497-2652

**Abstract-**

A diversity of aquatic organisms manage predation risk by avoiding waters activated with conspecific alarm cues, a chemical mixture released from injuries. The sea lamprey (*Petromyzon marinus*) is a nocturnal migratory species that relies on its alarm cue to navigate around areas of predation risk when moving through river channels. Identification of the cue’s chemistry would allow managers to harness this innate behavioral response to guide migrating sea lamprey to traps (invasive population in the Laurentian Great Lakes) or to fish passage devices where dams block migrations in their native range. We pursued isolation of the sea lamprey alarm cue through behaviorally guided fractionation, fractionating the alarm cue into water-soluble and chloroform-soluble fractions, each of which elicited a substantial avoidance response. Recombining the two fractions restored full reactivity, suggesting the alarm cue mixture contains components that exhibit high solubility in water (e.g., nitrogenous compounds), chloroform (e.g., lipids), or perhaps materials that dissolve readily in either solvent. We further screened 13 individual compounds or pure isolates and 6 sub-fractions from the water-soluble fraction and found one of the pure isolates, isoleucine, evoked an avoidance response on its own, but not consistently when found in other mixtures. In a third experiment, we observed no behavioral response after recombining 32 compounds isolated and identified from the water-soluble fraction. These results confirm other suggestions that the process of elucidating alarm cue constituents is challenging. However, we suggest the pursuit is worthwhile given the strong evidence for the utility of alarm cue for use in conservation and management of fishes and other aquatic organisms.

**Keywords-** Semiochemical; predation; risk; pheromone; behavioral manipulation; alarm substance

INTRODUCTION

Animals perceive and react to predation risk, and they balance the costs of their responses against other needs, including energy acquisition and reproduction (Ferrari, Sih, & Chivers, 2009; Lima & Bednekoff, 1999). Migratory species face a particular challenge, as movement between distant foraging and reproductive habitats requires individuals to navigate through complex risk landscapes where the location and identity of predators is uncertain, and the environmental cues that indicate safety may be misaligned with actual risk (Gallagher et al., 2017; Moore, 2018; Sabal et al., 2021). Consequently, accurate assessments of the immediacy of predation risk in space and time is crucial to migratory success.

The ability to perceive sensory cues associated with predation risk may be innate or acquired. Innate predator recognition is unlearnt and exhibited in a variety of prey organisms when they share an eco-evolutionary history with the predator, or a closely related species (Carthey & Blumstein, 2018). For example, the Seychelles warbler (*Acrocephalus sechellensis*) will respond to decoy predators whether born in populations isolated from predators or not (Veen et al., 2000), naïve giant pandas (*Ailuropoda melanoleuca*) display defense behaviors when exposed to predator urine (Du et al., 2012), and newly hatched Atlantic salmon (*Salmo salar*) show innate antipredator behaviors to piscivorous pike (*Esox lucius*) (Hawkins, Magurran, & Armstrong, 2004). Acquired predator recognition typically involves learning (Ferrari, Gonzalo, & Chivers, 2007). Examples include blue tits (*Cyanistes caeruleus*) and great tits (*Parus major*) that acquire recognition of acoustic predator cues through socially mediated learning (Keen et al., 2020), and zebrafish (*Danio rerio*) that can learn to label novel odors as risky when paired with known fear cues (Lucon-Xiccato et al., 2020).

Among aquatic organisms, these so-called “fear” cues include damage-released alarm cues. Alarm cues are public information; substances released from the tissues of injured conspecifics that reliably alert receivers to the presence of an active predator (Chivers & Smith, 1998; Smith, 1992; Wisenden, Vollbrecht, & Brown, 2004). Exposure to an alarm cue typically elicits antipredator behaviors including increased shelter use, decreased activity, and area avoidance (Lawrence & Smith, 1989; Ferrari et al., 2010; Wisenden, 2015). Evidence suggests alarm cues unite innate and acquired risk recognition. Detection of the alarm cue is innate (Lucon-Xiccato et al., 2020; Atherton & McCormick, 2015; Poisson et al., 2017), and patterns in the dispersion of the cue in the environment reveal locations of risk for conspecifics and closely related species who may share similar predators (Døving & Lastein, 2009; Ferrari, Wisenden, & Chivers, 2010; Hume & Wagner, 2018). When alarm cues are presented with the odor of an unfamiliar predator, the prey may learn to associate predator odor with danger and avoid it in the future (Brown, 2003; Ferrari, 2005; Kelley & Magurran, 2003). Alarm cue associated learning is important in the life history of settling coral reef fish, by facilitating predator detection during transitional life stages (Holmes & Mccormick, 2010), and pairing alarm cue odors with predator odors has been used to condition hatchery-reared fish to promote post-release survival (Griffin, 2004; Hawkins, Magurran, & Armstrong, 2008; Kopack et al., 2016; Sloychuk et al., 2016). This duality makes alarm cues particularly useful in mitigating uncertain risk landscapes during migration because alarm cue is consistently associated with a direct risk of injury to conspecifics or closely related species.

The sea lamprey (*Petromyzon marinus*) is a semelparous ectoparasitic fish that relies extensively on olfaction to complete its terminal spawning migration from the open waters of oceans or large lakes into streams. Migrants are guided into streams by the odor of conspecific larvae that labels the habitat as suitable for spawning and offspring survival (Sorensen, Vrieze, & Fine, 2004; Sorensen & Vrieze, 2003; Vrieze, Bergstedt, & Sorensen, 2011; Wagner et al., 2006; Wagner, Twohey, & Fine, 2009). Transition from deep open waters into narrow shallow streams exposes migrants to a suite of difficult to detect predators that patrol the shorelines (Imre et al., 2014; Scott & Crossman 1998; Boulêtreau et al., 2020). Because this migration is nocturnal, and sea lamprey move solitarily (Almeida, Quintella, & Dias, 2002; Binder & McDonald, 2007; McCann et al., 2018), they must rely on chemical public information to assess predation risk. Consequently, it is unsurprising that exposure to their alarm cue elicits immediate antipredator responses in rivers, including movement away from the shoreline activated with the cue (Hume et al., 2015; Imre et al., 2010; Wagner et al., 2011) and acceleration to pass through the risky area more quickly (Luhring et al. 2016).

Exploiting the sea lamprey’s behavioral responses to the alarm cue is driving the development of innovative approaches to control this species in the Laurentian Great Lakes where they are invasive, that also could be used to conserve them in locations where they are native (Imre et al., 2010; Wagner et al. 2022). For example, in the Great Lakes, traps are used to capture sea lamprey, and encounter rates with traps determine their effectiveness (Bravener & Mclaughlin, 2013; Miehls et al., 2020). Traps cannot be effectively baited, as sea lampreys cease feeding prior to the spawning migration, and attempts to bait with attractant pheromones have proven insufficient (Johnson, Siefkes, et al., 2015; Johnson et al., 2013; Johnson, Tix, et al., 2015). Application of the alarm cue to the opposite side of a river channel substantially increases encounter rates with traps placed near dams and in open river channels (Hume et al. 2015; Hume, Luhring, & Wagner, 2020). Within their native range, migrating sea lampreys are blocked from spawning habitat by dams (Hogg, Coghlan Jr, & Zydlewski 2013; Kynard & Horgan, 2019; Lasne et al., 2015). Here too, conservation outcomes could be improved by using the alarm cue to guide migrants toward fish passage devices (Byford et al., 2016; Hume et al., 2020; Pereira et al., 2017). Consequently, there is substantial interest in isolating and identifying the chemical constituents of the odor to support cost-effective synthesis of the large quantities needed for use of a repellent to achieve conservation goals, and to meet Federal requirements for pesticide registration (Ferguson & Gray, 1989).

Describing the chemical messengers that constitute fish alarm cues is a critical gap in our understanding of alarm cues and in being able to employ these for conservation or invasive species management (Døving & Lastein, 2009; Ferrari et al., 2010; Wisenden, 2000). Yet, few efforts have sought to identify compounds in fish alarm cues, and few commonalities in the compounds that may constitute the odors have arisen. For example, hypoxanthine-3-N-oxide (H3NO) has been hypothesized to be an active molecule in alarm cues from teleost fishes (Pfeiffer et al., 1984). Synthesized H3NO elicits consistent alarm responses in zebrafish (Parra, Adrian, & Gerlai, 2009), fathead minnows (Brown, Adrian, & Shih, 2001) and black tetra (Pfeiffer et al., 1984), but exhibited conflicting responses in salmonids and cichlids (Brown et al., 2003). This led to the suggestion that the nitrogen oxide functional group is important in initiating antipredator behavior, but is anchored to purine rings that differ in structure across taxa, allowing for species specificity in the cue (Brown et al., 2003). Another compound, chondroitin sulfate, elicits alarm responses in zebrafish (Mathuru et al., 2012) and fathead minnows (Faulkner et al., 2017), but the activity is less than the cue produced by injured tissue from the same species, suggesting the alarm cue is a mixture. One common pattern that has arisen is response to alarm cues obtained from closely related taxa, where the magnitude of the alarm response declines with increasing phylogenetic distance between the cue donor and the responding species (Mirza & Chivers, 2001; Mitchell, Cowman, & Mccormick, 2012; Schoeppner & Relyea, 2009; Mathis & Smith 1993). In previous studies, sea lamprey exhibit the phylogenetic relatedness pattern when responding to the cues from other lampreys, but did not respond to alarm cues extracted from bluegill sunfish (*Lepomis macrochirus*) or white sucker (*Catastomus commersoni*), suggesting little or no overlap between lampreys of the Petromyzontiformes and the distantly related clades in the Teleostei (Bals & Wagner 2012; Hume & Wagner 2018).

The most common method used to isolate olfactory cues in aquatic organisms is behaviorally guided fractionation, a stepwise iterative process that partitions an odor into fractions, typically by molecular weight, and uses a behavioral bioassay to identify the reactive fractions (Scott, Li, & Li, 2018). This process has been successful in the identification of key components used in chemical communication of aquatic and marine systems, including chemical defenses in common seaweed (*Lobophora variegata;* Kubanek et al., 2003) and reef sponges (*Erylus formosus;* Kubanek et al., 2000) , and sex pheromones in a variety of species (Algranati & Perlmutter, 1981; Yambe et al., 2006; Zielinski et al., 2004) including sea lamprey (Li et al., 2002; Scott et al., 2018). The chemistry of alarm cues has proven more enigmatic, with some species exhibiting reactivity to individual fractions, and others requiring all fractions from a crude odor extract in combination to elicit any antipredator response (Mirza, Laraby, & Marcellus, 2013). The aim of this study was to pursue the chemical constituents of the sea lamprey alarm cue using behaviorally guided fractionation. We examined the reactivity to two major subfractions of the full alarm cue extract (chloroform- and water-soluble) and examined responses to 32 compounds that have been previously identified from the highly reactive water-soluble fraction (Dissanayake, Wagner, & Nair, 2016, 2019), alone and in combination, in a standard laboratory assay.

METHODS AND MATERIALS

*Study Design.* To begin isolation of the alarm cue, we fractionated crude skin extract and tested the activity of individual sub-fractions and isolated compounds in a behavioral assay through a series of three experiments. The assay involves independent replicates, each consisting of a group of ten unique migratory sea lamprey used in only a single trial in a large raceway with a standard two-choice test where an odor is pumped into one side of flow and animals are free move throughout the arena. Avoidance or preference is ascertained from the distribution of fish on the two sides of the raceway (with or without the test odor), and the side receiving the odor (left or right) is alternated across replicates. A fish is used only once in a single replicate. The first experiment evaluated the sea lamprey’s behavioral response to a solvent control (N = 20), crude alarm cue extracts derived from the whole body (N = 20) or the skin (N = 20), a water-soluble (WS) fraction derived from the skin alarm cue (N = 20), a chloroform-soluble (CS) fraction derived from the skin extract (N = 20), and the WS and CS extracts combined (N = 20). Prior reports have indicated that the WS fraction from skin exhibited full behavioral reactivity when compared to whole-body extracts, with indications of partial and highly variable reactivity to the CS fraction (Dissanayake et al. 2019). The second experiment screened (sample sizes listed below) a series of six sub-fractions, 13 isolated compounds, and one compound mixture derived from the WS fraction to ascertain whether the behavioral reactivity was contained within one or a few sub-fractions. Another compound, chondroitin-sulfate, was not isolated from the WS extract, but was also screened, as previous studies found it played a role in the teleost fish alarm response (Farnsley et al., 2016; Faulkner et al., 2017; Mathuru et al., 2012). Because screening failed to identify a clear set of highly reactive candidate sub-fractions, the third experiment sought to determine whether partial or full reactivity was contained in the set of individual compounds that had been isolated and identified from these sub-fractions to date. We created a mixture of the 32 identified compounds that represented 98% (dry weight) of the material contained in the WS fraction and compared the behavioral reactivity of the mixture (N = 20) to the crude WS extract (N = 20) and solvent (N = 20).

*Odor Collection.*

***Whole body extract****-* Alarm cue was obtained from Soxhlet extraction of sea lamprey carcasses that naturally senesced in captivity per the method of Wagner et al. 2011. Odor extracted from recently deceased animals elicit alarm responses equivalent to those from live donors (Bals & Wagner 2012). In short, odor was derived through Soxhlet extraction from nine male and female sea lamprey weighing 1,496.5g total. All carcasses were kept at -20° C before being used in extractions. Soxhlet extractors (2.08m, Ace Glass Inc., Vineland, New Jersey, USA) were mounted to six-bulb water-cooled Allihn condensers. Solvent reservoirs (12L capacity) were loaded with 50:50 solution of 200 proof ethanol and deionized water and heated to 75 – 80° C with a hemispherical mantle for a minimum of three cycles (approximately six hours), creating ~10.2L of alarm cue extract. Extractions were cooled overnight before being decanted and filtered through muslin and were kept in a -20°C freezer until use in behavioral assays.

***Crude skin extract, fractionation, and identification of individual compounds****-* Experimental details of collection of alarm cue from sea lamprey skin, solvent-solvent partitioning of aqueous ethanolic skin extract in to chloroform-soluble and water-soluble fractions and MPLC fractionation (normal and reverse phase) of chloroform-soluble and water-soluble fractions are fully described in Dissanayake, Wagner, & Nair (2016, 2019, 2021). Purification of the MPLC subfractions of chloroform-soluble fraction was accomplished by preparative thin layer chromatography (Dissanayake et al., 2016). Purification of the MPLC subfractions of the water-soluble fraction was carried out by preparative HPLC (Dissanayake, Wagner, & Nair, 2019, 2021), respectively. The chemical identity of all pure isolates from the chloroform-soluble and water-soluble fractions was determined by NMR (1D and 2D) and HRESIMS experiments as described in Dissanayake, Wagner, & Nair (2016, 2019, 2021). Chondroitin sulfate used in experiment 2 screening was sourced from shark cartilage (Sigma-Aldrich, CAS-No. 9082-07-9).

***Mixture of known compounds in the water-soluble fraction***- We mixed the 32 previously identified compounds from the water-soluble fraction (Dissanayake et al., 2019) at observed ratios and concentrations found in the water-soluble fraction, based on mass (Table 2). Each compound (dry material) was weighed and then dissolved in 10 mL stock solvent solution (50:50 DI H_2_O: EtOH). Solutions were combined and brought up to final volume with solvent. The mixture was refrigerated until use, within 48 h.

*Test Subjects.* All sea lamprey used in experiments were migratory sub-adults obtained via the U.S. Fish and Wildlife Service’s trapping operations in the Cheboygan and Ocqueoc Rivers (tributaries to Lake Huron in Michigan, USA), and the St. Mary’s River connecting channel between Lakes Superior and Huron. Actively migrating sea lamprey were collected in large traps arrayed near dams and transported to the Hammond Bay Biological Station (HBBS) in tanks receiving continuous aeration. Fish were sorted by sex and held in 1385 L round tanks that received a continuous flow of Lake Huron water (100% exchange every 4 h) with supplemental aeration until use. Fish were held under natural day-night light cycles. Only males were used in the study as female lamprey decrease their reactivity to alarm cue during sexual maturation, whereas males do not (Bals & Wagner, 2012). Prior studies with sexually immature migrants indicated no difference in response to alarm cue between sexes (Bals & Wagner, 2012). All animal procedures were approved by the Michigan State University Institutional Animal Care and Use Committee via permits AUF 02/16-015-00 and PROTO201900060.

*Behavioral Assay.* Experimental trials were conducted in two laboratory raceways at HBBS (Figure 1). Each raceway measured 1.44m x 12.2m, with a 3.1 m long reach isolated with block nets to form the experimental arena. The experimental arenas were lined with white plastic paneling (1/16in PLAS-TEX, Parkland Plastics, Inc., Middlebury, Indiana, USA) to increase visual contrast between lampreys and their background. Experiments took place in full darkness and were recorded with overhead infrared sensitive video cameras (Axis Communications, Q1604 Network Camera), each illuminated by an array of six infrared lights (Wildlife Engineering; Model IRLamp6). Water flowed into flumes from a head tank supplied directly from Lake Huron. Water temperature ranged from 6-18 °C over the course of trials, in accordance with seasonal changes in lake temperature. Discharge was maintained at 0.02-0.03 m^3^ sec^-1^ in each channel.

Because the sea lamprey is a nocturnal migrant, all trials were conducted between 18:00 and 02:00 hours during the spring migratory season. Two hours before experimental trials began subjects were visually inspected to ensure immature status and transferred to holding baskets with ten animals per basket, constituting trial groups. Each trial group represented a single independent sample, and each fish was only used once in a single trial. As such, each independent trial group represented a single replicate, with 1,200 fish total used for experiment 1, 1,800 fish total used for experiment 2, and 600 fish used for experiment 3. Each trial began by carefully releasing the ten animals from their holding basket into the middle of the experimental arena. Trials lasted 30 min including a 10 min acclimation period and a 20 min observation period, during which test odors were introduced. During a trial, test odors were introduced into one-half of the experimental arena (left or right side), with the side receiving the odor alternating on subsequent replicates. All odors were pumped into the channels from a beaker at the rate necessary to achieve a 1 000 000:2 DI water:odor extract dilution. To achieve this dilution, 88mL of odor extract was placed in a beaker and brought up to a total 524mL odor solution by adding 436mL of DI water (calculations of odor extract dilutions were done based on activated channel width, depth, and velocity). The odor solution was then transferred to a 1L Nalgene bottle and continuously stirred with a 2cm magnetic stir bar to ensure a homogenous mixture. Odors were pumped into the system at a fixed rate of 20mL min^-1^ with peristaltic pumps (MasterFlex model 7533-20) through PVC tubing. A separate set of tubing was used for each odor or odorant to ensure no cross contamination occurred. Visual rhodamine dye tests were conducted to confirm the odor plume was confined to the target half of the experimental arena. At the conclusion of each trial, subjects were removed from the arenas and total length (TL, cm) and wet weight (g) were recorded for each individual.

*Analyses.*

***Video analysis****-* Behaviors were quantified for each trial, representing independent samples, during the final 10 min of the observation period (post-stimulus period) to ensure the odor reached the end of the raceway and allow for the distribution of animals to stabilize after the addition of the odor (approximately 5 min per Wagner et al., 2011). Each video recording was examined by pausing each 30s and tallying the number of fish on each side of the channel (stimulus side or non-stimulus side), based on position of the head, as an indication of channel preference. Distribution (proportion on the stimulus side) was calculated as follows:

#

$$\frac{\boldsymbol{\Sigma}\left( \boldsymbol{number} \boldsymbol{of} \boldsymbol{fish} \boldsymbol{on} \boldsymbol{the} \boldsymbol{stimulus} \boldsymbol{side} \right)}{\boldsymbol{\Sigma}\left( \boldsymbol{number} \boldsymbol{of} \boldsymbol{fish} \boldsymbol{on} \boldsymbol{the} \boldsymbol{stimulus} \boldsymbol{side} \right)\boldsymbol{+}\boldsymbol{\Sigma}\boldsymbol{(}\boldsymbol{number} \boldsymbol{of} \boldsymbol{fish} \boldsymbol{on} \boldsymbol{the} \boldsymbol{non}\boldsymbol{-}\boldsymbol{stimulus} \boldsymbol{side}\boldsymbol{)}}$$

The distribution for each treatment was computed by taking the mean of all trials in each treatment group. A proportion of fish significantly greater than 50% on the stimulus side indicated attraction, a proportion not significantly different from 50% indicated no preference, and a proportion significantly less than 50% indicated avoidance.

***Statistical analysis***- All analyses were conducted in R (Version 1.4.1103). A one-way ANOVA was performed for each experiment with response variable as proportion of animals on the stimulus side and stimulus (odor) type as fixed effect. In experiment 1, data were transformed using log transformations and normality was confirmed with a Shapiro-Wilk’s test (α = 0.05). Tukey’s Honestly Significant Difference (HSD) (α = 0.05) was completed as a post-hoc means comparisons for each treatment. The mean proportion of fish on the stimulus side for the water-soluble and chloroform-soluble fractions were compared to the whole body and crude skin alarm cue extracts, and the solvent control, to determine whether the alarm cue was partially, completely, or not significantly contained in either fraction. In experiment 2, data were transformed with an arcsine (square root) transformation and normality was confirmed with a Shapiro-Wilk’s test (α = 0.05). The means of a whole-body extract treatment, solvent treatment, 14 individual compounds, one mixture of individual compounds, and 6 subfractions (Table 1) were compared to a null hypothesis of 50:50 proportion of fish on the stimulus side with separate paired t-tests for each odor (two-tailed, α = 0.05) to screen for any attractant or repulsive response. In experiment 3, data were log transformed and normality was confirmed with Shapiro-Wilk’s test (α = 0.05). Tukey’s Honestly Significant Difference (HSD) (α = 0.05) was used for post-hoc means comparisons of each treatment. Here, the mean of the mixture of the identified compounds from the water-soluble fraction was compared to the whole-body alarm cue extract, the water-soluble fraction, and the solvent control to determine whether the alarm cue was contained within these identified components.

RESULTS

*Experiment 1: Comparison of Water- and Chloroform- Soluble Fractions.* Model results (ANOVA, *F*_5,114_ = 12.76, *p* < 0.001) showed clear evidence that the type of odor introduced into the raceway significantly influenced the sea lamprey’s use of space. Both alarm cue extracts (skin, whole-body) showed significant avoidance when compared to the solvent control (Tukey HSD, all solvent comparisons *p* < 0.05; Figure 2). The response to the whole-body extract was not significantly different from that of the crude skin extract (Tukey HSD, *p* = 0.99; Figure 2). Avoidance of the water-soluble fraction was 33% less than the whole-body extract (Tukey HSD, *p* < 0.01; Figure 2) and 28% lower than the crude skin extract (Tukey HSD, *p* < 0.05; Figure 2), and was not significantly different from the chloroform-soluble fraction (Tukey HSD, *p* = 0.98; Figure 2). Avoidance of the chloroform-soluble fraction was not significantly different from the whole-body extract (Tukey HSD, *p* = 0.05; Figure 2), the crude skin extract (Tukey HSD, *p* = 0.18; Figure 2), or the water-soluble fraction (Tukey HSD, *p* = 0.98; Figure 2). The behavioral response to the water-soluble fraction appeared more consistent (variance = 0.006; Figure 2) than the response to the chloroform-soluble fraction (variance = 0.02; Figure 2). Avoidance of a mixture of the water-soluble and chloroform-soluble fractions was not significantly different than observed for the whole-body extract (Tukey HSD, *p* = 0.99; Figure 2) or the crude skin extract (Tukey HSD, *p* = 0.99; Figure 2)

*Experiment 2: Screening of Sub-Fractions and Compounds in the Water-Soluble Fraction.* Odor introduced into the channel significantly influenced sea lamprey behavior in the screenings of individual compounds and subfractions (ANOVA, F_22,173_ = 2.12, *p* < 0.01). As predicted, the observed response to the solvent control was not significantly different from the null expectation of a 50:50 distribution (*t*_19_ =0.36, *p* = 0.73, Table 1), and subjects significantly avoided the whole-body alarm cue (*t*_36_ = -6.21, *p* < 0.001, Table 1). Only one of the 13 individual compounds screened from the water-soluble fraction elicited significant avoidance, isoleucine (*t*_9_ = -2.47, *p* = 0.04, Table 1). Another compound, hypoxanthine, exhibited a marginally non-significant avoidance response (*t*_9_ = -2.13, *p* = 0.06, Table 1). Similarly, only one of the six screened subfractions, SL-4, elicited avoidance (*t*_4_ = -3.15, *p* = 0.03, Table 1), and contained creatine, arginine, valine, and isoleucine. Other than isoleucine, none of these compounds showed an avoidance response when tested alone (creatine, *t*_4_ = -0.09, *p* = 0.93; arginine, *t*_4_ = -0.90, *p* = 0.42; valine, *t*_4_ = 0.37, *p* = 0.73; Table 1). Additionally, a mixture of isoleucine, tyrosine, and hypoxanthine (*t*_4_ = -0.15, *p* = 0.89, Table 1) showed no evidence of behavioral reactivity. Chondroitin-sulfate also showed no evidence of behavioral reactivity (*t*_4_ = 1.06, *p* = 0.35, Table 1).

*Experiment 3: Testing the Mixture of Identified Compounds from the Water-Soluble Fraction.* Odorant type significantly influenced sea lamprey avoidance behavior (ANOVA, *F*_2,58_ = 8.99, *p* < 0.001).

Here, a mixture of the 32 identified compounds from the water-soluble fraction exhibited no avoidance response, indicated by no significant difference in response when compared to the negative solvent control (Tukey HSD, *p* = 0.59; Figure 3), and a significantly lower avoidance response compared to the water-soluble fraction (Tukey HSD, *p* < 0.001; Figure 3).

DISCUSSION

Recent research has revealed several potential uses for the sea lamprey alarm cue as a species-specific repellent that can aid in the management of both invasive and threatened populations. We report the behavioral responses of migratory sea lamprey to two major odor fractions derived from Soxhlet extraction of the skin; a tissue known to contain the animal’s alarm cue. We found that both the water-soluble and chloroform-soluble fractions elicited substantial avoidance responses, with the water-soluble fraction exhibiting a significantly lower avoidance response than the crude skin extract and the chloroform-soluble fraction exhibiting an avoidance response no different than either the water-soluble or the crude skin. When the two fractions were recombined, the full response was restored. There were six sub-fractions derived from the water-soluble fraction, from which 32 compounds were isolated and identified, representing 98% of the dry mass of extracted material. Only one individual compound, isoleucine, evoked an avoidance response during initial screening; however, this was not consistent across all treatments containing the compound. Finally, to test for synergistic effects, we examined the behavioral response of sea lamprey to a mixture of the 32 identified compounds combined at the ratios and quantities observed in the water-soluble fraction. The mixture failed to evoke an alarm response. Together, these results indicate that the active components of the sea lamprey alarm cue are contained in two chemically dissimilar fractions from skin but were not fully contained in the mixture of compounds identified to date.

Consistent with previous reports (Byford et al., 2016; Hume et al., 2015; Hume & Wagner, 2018; Imre et al., 2014; Imre et al., 2016; Luhring et al., 2016; Wagner et al., 2016; Wagner, Stroud, & Meckley, 2011), we observed a predator avoidance response to extracts from sea lamprey skin tissue that is consistent with the hypothesis that certain components of the cue exhibit substantial water solubility and are nonvolatile. Specifically, the water-soluble fraction of Soxhlet-extracted skin invoked 72% of the avoidance response observed from the crude extract.

Sea lamprey did not exhibit consistent avoidance responses to the 13 individual compounds, one compound mixture, or six sub-fractions from the water-soluble fraction subset in the screening experiment (Experiment 2, Table 1). While isoleucine exhibited an avoidance response on its own, other treatments where it was mixed with other compounds (namely tyrosine and hypoxanthine), exhibited no avoidance response (Table 1). One possibility is that tyrosine or hypoxanthine could act as an antagonist for isoleucine. However, it has been shown that leucine and valine act antagonistically with isoleucine (amino acids found in the avoidant SL-4 fraction), whereas tyrosine (found in the neutral mixture of isoleucine, tyrosine and hypoxanthine) does not (Kajikawa et al., 2005). It is also possible that the compounds were run at a sample size too small to accurately detect a significant response. Thus, none of the compounds that were evaluated was sufficient on its own to elicit consistent predator avoidance across all included treatments, but further investigation on the role of isoleucine at higher replication is needed to understand if it elicits a robust avoidance response.

When all 32 identified compounds from the water-soluble fraction (Experiment 3, Table 2) were combined at the observed ratio found in the crude skin extract, a neutral response similar to the solvent treatment was observed (Figure 3). One plausible explanation for why we saw no antipredator responses to individual compounds, sub-fractions, or recombined identified compounds within the water-soluble fraction is that the alarm cue may consist of a blend of compounds, all of which need to be present in order to elicit a behavioral response. Previous studies have noted singular compounds can be potent in eliciting alarm responses and are hypothesized to contain a component of the active ingredients of alarm cue, such as hypoxanthine 3-N-Oxide in zebrafish (Parra et al., 2009), fathead minnows (Brown, Adrian, & Shih, 2000), and black tetra (Pfeiffer et al., 1984), and chondroitin sulfate in zebrafish (Mathuru et al., 2012) and fathead minnows (Faulkner et al., 2017). Other studies suggest the full mixture needs to be present (larval grey tree frog *Hyla vesicolor*, Mirza et al., 2013), sea hare *Aplysia californica,* Kicklighter et al., 2007)). Our findings, along with the observed diminishing reactivity with increasing phylogenetic distance between donor and receiver in sea lamprey (Bals and Wagner 2012; Hume and Wagner 2018) align to suggest the sea lamprey alarm cue is a mixture of active components, with some shared compounds across species and species-specific labeling compounds (i.e., the multicomponent pheromone hypothesis). Another possible explanation is that the active compounds of the alarm cue degraded or were otherwise lost during the separation and purification process. Extractions of chemical defense compounds from one vascular plant (*Micranthemum umbrosum*) led to significant degradation and inefficient yields, which may have degraded potentially active compounds below a detection threshold (Parker et al. 2006). In the present study, bioassay guided isolation and purification were conducted under mass balance at every step, and there was no noticeable loss of material during the purification based on mass balance of the original extract or fraction to the isolated compounds. However, given the lack of activity elicited by the major components of the active fractions, the alarm cue components may reside in the 2% of unidentified material remaining in the crude extract. It is also possible that the process of separation and isolation led to degradation of the active molecules after extraction if components in the crude extract functioned as stabilizers for the active material. Future research should focus on identifying the minor compounds of the water-soluble fraction to understand if they complete the cue and play a role in mediating antipredator behavior.

The compounds identified from the water-soluble fraction consisted of 32 amino acids, primarily creatine (Dissanayake et al., 2019). Green et al. (2017) demonstrate that all regions of the sea lamprey olfactory bulb respond to amino acids, with the lateral bulb responding solely to amino acids and the dorsal and medial regions responding dually to amino acids and steroids, noting that these regions do not act redundantly but rather react to different types of information. Amino acids are associated with feeding behavior in sea lamprey (Kleerekoper and Mogenson, 1963). The concentration of combined amino acids from the water-soluble fraction was 0.023 mol l^-1^, and the range of individual compounds between 10^-5^ mol l^-1^ and 0.536 mol l^-1^. All but six amino acids in the extract were individually above the probable threshold of detection for sea lamprey (10^-3^ mol l^-1^, (Green et al., 2017)). Putrescine (10^-4^ mol l^-1^), pyruvic acid (10^-4^ mol l^-1^), serine (10^-5^ mol l^-1^), adenosine (10^-5^ mol l^-1^), spermine (10^-5^ mol l^-1^), and α-ketovaleric acid (10^-5^ mol l^-1^), were below this threshold. The molarity of the recombined water-soluble fraction should therefore be well above the detection threshold. Migratory sea lamprey are non-feeding, relying on lipids stored during the parasitic life stage to spawn and complete their lifecycle before death (William & Beamish, 1979). It would be reasonable for responses to food cues to cease prior to the spawning migration to focus olfactory efforts on avoiding predation and finding mates. Life-stage dependent olfactory sensitivity has been cited in the Pacific lamprey, where reactivity to migratory and sex pheromones remained high and constant throughout the spawning migration before dropping significantly at spawning and maturation, and it is noted that Pacific and sea lampreys share remarkable similarities in odor responses both ecologically and physiologically (Robinson et al., 2009) .

We observed a substantial avoidance response to the chloroform-soluble fraction that was similar in magnitude to the water-soluble fraction, but statistically indistinguishable from the crude extract due to higher variance in the behavioral responses. This finding contrasts somewhat with Dissanayake et al. (2016) who reported a partial (61% of the crude skin extract response) but statistically non-significant avoidance response to a similar fraction. A balanced one-way ANOVA power calculation for the data in Dissanayake et al. (2016) revealed that a sample size greater than or equal to 17 was needed to detect a significant response (power = 0.80, α = 0.05), which was surpassed in the current study (N = 20), but not in the 2016 screening (N = 10). There are at least two plausible explanations for these observations. First, the chloroform-soluble fraction may contain one or more components of the alarm cue that are reactive and not found in the water-soluble fraction at concentrations sufficient to be detected by the olfactory organ. The major components of this fraction were previously identified as four cholesterol esters, five tri- and di-glycerides, a cholesterol, 13 free fatty acids, and two environmental pollutants (Dissanayake et al., 2016), but not tested individually for behavioral responses. Fatty acids have been shown to be behaviorally relevant in migrating sea lamprey; an active compound, (+)-petromyric acid, of the attractant cue emitted by larvae is a fatty-acid derivative (Li et al., 2018). More broadly, three olfactory sensory neuron (OSN) morphotypes have been identified in teleost fishes (Hamdani & Døving, 2007), and the structure of these morphotypes are strikingly similar to those observed in the more primordial sea lamprey (Laframboise et al., 2007). In teleost fishes, the ciliated OSN activates the medial olfactory tract and responds to compounds important in both migration and alarm responses (Hamdani & Døving, 2007; Døving & Lastein, 2009). The medial bulbar region of the sea lamprey olfactory bulb responds to amino acids, bile salts, and components of the larval cue (Green et al., 2017). If the overlap in sensory pathways among migratory and alarm cues is present in lampreys, further testing of OSN pathway activation may help to discern the identity of the alarm cue component(s) contained in the chloroform-soluble fraction.

Reactivity in the chloroform-soluble fraction could also be attributed to incomplete separation of the mixture, with one or more behaviorally reactive compounds occurring in both major fractions. Interestingly, when the water-soluble and chloroform-soluble fractions were re-combined, the magnitude of the avoidance response increased to that observed from the crude skin extract. Recombination may have restored the correct ratios of alarm cue compounds. Studies on alarm cue phylogenetic patterning in Ostariophysan fishes have suggested that reactivity is dependent on observed ratios of compounds, and that such ratios are species-specific. For example, the purine ratio hypothesis posits the existence of a common set of purine carriers for a nitrogen-oxide alarm trigger in Ostariophysan fishes, with ratios of the carrier molecules differing among related species, and larger differences between more distantly related species (Brown et al., 2001; Brown et al., 2003; Kelly et al., 2006). Alternatively, or in addition, recombination may have restored the full concentration of alarm cue components, eliciting a stronger behavioral reaction. The threat-sensitive response hypothesis predicts that prey who modulate their antipredator behavior in response to the perceived intensity of the threat will have a selective advantage (Helfman, 1989). Fishes (Brown et al., 2006; Lönnstedt & Mccormick, 2011), amphibians (Ferrari et al., 2009; Fraker, 2008), and aquatic insects (Roux & Diabate, 2014) are known to respond to varying concentrations of alarm and predator cues in a threat-sensitive manner.

Previous studies have demonstrated that chondroitin fragments play an active role in fish alarm cue chemistry (Faulkner et al., 2017; Mathuru et al., 2012). During screening, sea lamprey failed to respond to chondroitin sulfate derived from shark cartilage (Table 1). An intermediate alarm response was observed in wild fathead minnows (*Pimephales promelas*) that were introduced to chondroitin sulfate sourced from bovine trachea (Faulkner et al., 2017). Zebrafish exhibited a full suite of alarm behaviors when exposed to chondroitin sulfate sources from shark cartilage and an intermediate response when exposed to chondroitin sulfate from sturgeon notochord, and these differences are likely due to source differences in sulfation which affects signaling properties of chondroitin (Mathuru et al., 2012). Because of the observed differences in fish response to chondroitin sulfate sources, more research into the sea lamprey behavioral response to differently sulfated forms of chondroitin may be warranted. However, as noted above, given the apparent lack of response by sea lamprey to alarm cues of teleost fishes, they may be chemically distinct.

In summary, this study represents the first major steps towards identifying the sea lamprey alarm cue. Our work provides evidence in support of previous studies that hypothesized the sea lamprey alarm cue contains a mixture of stable molecules (Bals & Wagner, 2012; Dissanayake et al., 2019; Hume & Wagner, 2018), and suggests for the first time that the active constituents are not solely contained in the water-soluble fraction of the crude skin extract. Thus, questions remain regarding the active components of the sea lamprey alarm cue. Future work should include investigating the role of isoleucine and focus on discovering the identity of minor compounds within the water-soluble fraction, testing individual and combined compounds identified from the chloroform-soluble fraction, targeting areas of potential overlap. Additional research is needed to explore the evolution of olfactory roles in predator-prey dynamics, and to understand how alarm cues can be synthesized and thus applied towards conservation goals and the management of aquatic invasive species. We argue that the pursuit of the alarm cue’s chemical identity is crucial to answer questions on the evolution of chemosensory cues in predator-prey dynamics and can lead to important information on how wildlife managers and conservation professionals can use such cues for applied work in aquatic systems.

ACKNOWLEDGEMENTS

Funding for this project was provided by the Great Lakes Fishery Commission. Thank you to staff at US Geological Survey’s Hammond Bay Biological Station for providing access to the raceway laboratory, supplying sea lamprey, and technical aid. We give special thanks to Dr. Nicholas Johnson and Christopher Wright for creating safety protocols which allowed us to work safely at HBBS during the Covid-19 pandemic. Mikaela Hanson, Kandace Griffin, Taylor Haas, Anna Tursky, Madison Perry, Kristopher Rygiel, Emily Suominen, Grace Forthaus, Tyler Welklin, Sydney Tieman, Allie Yackley, Michael Nolan-Tamariz, and Alana Barton provided invaluable laboratory and video analysis assistance. We thank Dr. Brian Roth and Dr. Cindy Baker for their helpful feedback on the study design and interpretation of results. Two anonymous reviewers provided thoughtful feedback that improved the manuscript prior to publication.

REFERENCES

Algranati FD & Perlmutter A (1981) Attraction of zebrafish, *Brachydanio rerio*, to isolated and partially purified chromatographic fractions. Env Biol Fish 6(1): 31–38.

Almeida PR, Quintella BR & Dias NM (2002) Movement of radio-tagged anadromous sea lamprey during the spawning migration in the River Mondego (Portugal). Hydrobiologia 483:1–8.

Atherton JA & McCormick MI (2015) Active in the sac: Damselfish embryos use innate recognition of odours to learn predation risk before hatching. Anim Behav 103: 1–6. https://doi.org/10.1016/j.anbehav.2015.01.033

Bals JD & Wagner CM (2012) Behavioral responses of sea lamprey (*Petromyzon marinus*) to a putative alarm cue derived from conspecific and heterospecific sources. Behaviour 149(9): 901–923. <https://doi.org/10.1163/1568539X-00003009>

Binder TR & McDonald DG (2007) Is there a role for vision in the behaviour of sea lampreys (*Petromyzon marinus*) during their upstream spawning migration? Can J Fish Aquat Sci 64(10): 1403–1412. https://doi.org/10.1139/F07-102

Boulêtreau S, Carry L, Meyer E, Filloux D, Menchi O, Mataix V, & Santoul F (2020) High predation of native sea lamprey during spawning migration. Sci Rep 10: 1–9. https://doi.org/10.1038/s41598-020-62916-w

Bravener GA & McLaughlin RL (2013) A behavioural framework for trapping success and its application to invasive sea lamprey. Can J Fish Aquat Sci 70: 1438–1446. https://doi.org/10.1139/cjfas-2012-0473

Brown GE, Adrian JC & Shih ML (2001) Behavioural responses of fathead minnows to hypoxanthine‐3‐N‐oxide at varying concentrations. J Fish Biol 68: 1465–1470. https://doi.org/10.1006/jfbi.2000.1541

Brown GE, Adrian JC, Naderi NT, Harvey MC & Kelly JM (2003) Nitrogen oxides elicit antipredator responses in juvenile channel catfish, but not in convict cichlids or rainbow trout: conservation of the ostariophysan alarm pheromone. J Chem Ecol 29(8): 1781–1796.

Brown GE (2003) Learning about danger: chemical alarm cues and local risk assessment in prey. Fish Fish 4: 227–234.

Brown GE, Rive AC, Ferrari MCO & Chivers DP (2006) The dynamic nature of antipredator behavior: prey fish integrate threat-sensitive antipredator responses within background levels of predation risk. Behav Ecol Sociobiol 61: 9–16. https://doi.org/10.1007/s00265-006-0232-y

Byford GJ, Wagner CM, Hume JB & Moser ML (2016) Do native pacific lamprey and invasive sea lamprey share an alarm cue? Implications for use of a natural repellent to guide imperiled pacific lamprey into fishways. N Am J Fish Manag 36(5): 1090–1096. <https://doi.org/10.1080/02755947.2016.1198286>

Carthey AJR, Blumstein DT (2018) Predicting predator recognition in a changing world. Trends Ecol Evol 33:106–115. https://doi.org/10.1016/j.tree.2017.10.009

Chivers DP & Smith RJF (1998) Chemical alarm signaling in aquatic predator-prey systems: A review and prospectus. Ecoscience 5(3): 338–352.

Dissanayake AA, Wagner CM & Nair MG (2021) Evaluation of health benefits of sea lamprey (*Petromyzon marinus*) isolates using in vitro anti-inflammatory and antioxidant assays. PLoS One. https://doi.org/10.1371/journal.pone.0259587

Dissanayake AA, Wagner CM & Nair MG (2016) Chemical characterization of lipophilic constituents in the skin of migratory adult sea lamprey from the Great Lakes Region. PLoS One. https://doi.org/10.1371/journal.pone.0168609

Dissanayake AA, Wagner CM & Nair MG (2019) Nitrogenous compounds characterized in the deterrent skin extract of migratory adult sea lamprey from the Great Lakes region. PLoS One. https://doi.org/10.1371/journal.pone.0217417

Døving KB & Lastein S (2009) The alarm reaction in fishes - odorants, modulations of responses, neural pathways. Ann NY Acad Sci 1170: 413–423. https://doi.org/10.1111/j.1749-6632.2009.04111.x

Du Y, Huang Y, Zhang H, Li D, Yang B, Wei M, Zhou Y & Liu Y (2012) Innate Predator Recognition in Giant Pandas. Zool Sci 29(2): 67–70. https://doi.org/10.2108/zsj.29.67

Farnsley S, Kuhajda B, George A & Klug H (2016) *Fundulus catenatus* (Northern Studfish) response to the potential alarm cue chondroitin sulfate. Southeast Nat 15(3): 523–533.

Faulkner AE, Holstrom IE, Molitor SA, Hanson ME, Shegrud WR, Gillen JC, Willard SJ & Wisenden BD (2017) Field verification of chondroitin sulfate as a putative component of chemical alarm cue in wild populations of fathead minnows (*Pimephales promelas*). Chemoecology 27(6): 233–238. https://doi.org/10.1007/s00049-017-0247-z

Ferguson S & Gray E (1989) 1988 FIFRA Amendments: A Major Step in Pesticide Regulation. Envtl L Rep News & Analysis 19: 10070.

Ferrari MCO (2005) The role of learning in the development of threat-sensitive predator avoidance by fathead minnows. Anim Behav 70(4):777–784. https://doi.org/10.1016/j.anbehav.2005.01.009

Ferrari MCO, Gonzalo A & Chivers DP (2007) Generalization of learned predator recognition: an experimental test and framework for future studies. Proc R Soc B: Biol Sci 274(1620): 1853–1859. https://doi.org/10.1098/rspb.2007.0297

Ferrari MCO, Sih A & Chivers DP (2009) The paradox of risk allocation: a review and prospectus. Anim Behav 78(3): 579–585. https://doi.org/10.1016/j.anbehav.2009.05.034

Ferrari MCO, Wisenden BD & Chivers DP (2010) Chemical ecology of predator – prey interactions in aquatic ecosystems: a review and prospectus. Can J Zool 88: 698–724.

Fraker ME (2008) The dynamics of predation risk assessment: responses of anuran larvae to chemical cues of predators. J Anim Ecol 77(4):638–645. https://doi.org/10.1111/j.1365-2656.2008.01386.x

Frommen JG, Mehlis M & Bakker TCM (2009) Predator-inspection behaviour in female three-spined sticklebacks *Gasterosteus aculeatus* is associated with status of gravidity. J Fish Biol 75(8): 2143-2153. https://doi.org/10.1111/j.1095-8649.2009.02408.x

Gallagher AJ, Creel S, Wilson RP & Cooke SJ (2017) Energy landscapes and the landscape of fear. Trends Ecol Evol 32(2): 88–96. http://dx.doi.org/10.1016/j.tree.2016.10.010

Green WW, Boyes K, Mcfadden C, Daghfous G, Auclair F, Zhang, H, Li W, Dubuc R & Zielinski BS (2017) Odorant organization in the olfactory bulb of the sea lamprey. J Exp Biol 220: 1350–1359. https://doi.org/10.1242/jeb.150466

Griffin AS (2004) Social learning about predators: a review and prospectus. Learn Behav 32(1): 131-140.

Hamdani EH & Døving KB (2007) The functional organization of the fish olfactory system. Prog Neurobiol 82(2): 80–86. https://doi.org/10.1016/j.pneurobio.2007.02.007

Hawkins LA, Magurran AE & Armstrong JD (2004) Innate predator recognition in newly-hatched Atlantic salmon. Behaviour 141(10): 1249–1262.

Hawkins LA, Magurran AE & Armstrong JD (2008) Ontogenetic learning of predator recognition in hatchery-reared Atlantic salmon, *Salmo salar*. Anim Behav 75: 1663–1671. https://doi.org/10.1016/j.anbehav.2007.10.019

Helfman GS (1989) Threat-sensitive predator avoidance in damselfish-trumpetfish interactions. Behav Ecol Sociobiol 24: 47–58.

Hogg R, Coghlan Jr SM & Zydlewski J (2013) Anadromous sea lampreys recolonize a Maine coastal river tributary after dam removal. Trans Am Fish Soc 142(5): 1381–1394. https://doi.org/10.1080/00028487.2013.811103

Holmes TH & McCormick MI (2010) Smell, learn and live: the role of chemical alarm cues in predator learning during early life history in a marine fish. Behav Process 83(3): 299–305. https://doi.org/10.1016/j.beproc.2010.01.013

Hume JB, Lucas MC, Reinhardt U, Hrodey PJ & Wagner CM (2020) Sea lamprey (*Petromyzon marinus*) transit of a ramp equipped with studded substrate: Implications for fish passage and invasive species control. Ecol Eng 155: 1–11. https://doi.org/10.1016/j.ecoleng.2020.105957

Hume JB, Meckley TD, Johnson NS, Luhring TM, Siefkes MJ & Wagner CM (2015) Application of a putative alarm cue hastens the arrival of invasive sea lamprey (*Petromyzon marinus*) at a trapping location. Can J Fish Aquat Sci 72(12): 1799–1806. dx.doi.org/10.1139/cjfas-2014-0535

Hume JB & Wagner CM (2018) A death in the family: Sea lamprey (*Petromyzon marinus*) avoidance of confamilial alarm cues diminishes with phylogenetic distance. Ecol Evol 8(7): 3751–3762. https://doi.org/10.1002/ece3.3930

Imre I, Di Rocco RT, Belanger CF, Brown GE & Johnson NS (2014) The behavioural response of adult *Petromyzon marinus* to damage-released alarm and predator cues. J Fish Biol 84: 1490–1502. https://doi.org/10.1111/jfb.12374

Imre I, Di Rocco RT, Brown GE & Johnson NS (2016) Habituation of adult sea lamprey repeatedly exposed to damage-released alarm and predator cues. Environ Biol Fishes 99(8): 613–620. https://doi.org/10.1007/s10641-016-0503-z

Imre I, Brown GE, Bergstedt RA & McDonald R (2010) Use of chemosensory cues as repellents for sea lamprey: Potential directions for population management. J Great Lakes Res 36(4): 790–793. https://doi.org/10.1016/j.jglr.2010.07.004

Johnson NS, Siefkes MJ, Wagner CM, Bravener G, Steeves T, Twohey M & Li W (2015) Factors influencing capture of invasive sea lamprey in traps baited with a synthesized sex pheromone component. J Chem Ecol 41(10): 913–923. https://doi.org/10.1007/s10886-015-0626-2

Johnson NS, Siefkes MJ, Wagner CM, Dawson H, Wang H, Steeves T, Twohey M & Li W (2013) A synthesized mating pheromone component increases adult sea lamprey (*Petromyzon marinus*) trap capture in management scenarios. Can J Fish Aquat Sci 70: 1101–1108. dx.doi.org/10.1139/cjfas-2013-0080

Johnson NS, Tix JA, Hlina BL, Wagner CM, Siefkes MJ, Wang H & Li W (2015) A sea lamprey (*Petromyzon marinus*) sex pheromone mixture increases trap catch relative to a single synthesized component in specific environments. J Chem Ecol 41(3): 311–321. https://doi.org/10.1007/s10886-015-0561-2

A

Kajikawa H, Mitsumori M, Tajima K & Kurihara M (2005) Amino acids antagonistic to the amino acids inhibitory for growth rate of mixed ruminal bacteria. J Dairy Sci 88(7): 2601–2603.

Keen SC, Cole EF, Sheehan MJ & Sheldon BC (2020) Social learning of acoustic anti-predator cues occurs between wild bird species. Proc R Soc B: Biol Sci 287: 1-9. http://dx.doi.org/10.1098/rspb.2019.2513

Kelley JL & Magurran AE (2003) Learned predator recognition and antipredator responses in fishes. Fish Fish 4: 216–226.

Kelly JM, Adrian JC & Brown GE (2006) Can the ratio of aromatic skeletons explain cross-species responses within evolutionarily conserved Ostariophysan alarm cues?: testing the purine-ratio hypothesis. Chemoecology 16: 93–96. https://doi.org/10.1007/s00049-005-0333-5

Kicklighter CE, Germann M, Kamie M & Derby CD (2007) Molecular identification of alarm cues in the defensive secretions of the sea hare *Aplysia californica*. Anim Behav 74: 1481–1492. https://doi.org/10.1016/j.anbehav.2007.02.015

Kleerekoper H, Mogensen J (1963) Role of olfaction in the orientation of *Petromyzon marinus. I.* Response to a single amine in prey’s body odor. Physiol Zool 36: 347–360.

Kopack CJ, Broder ED, Fetherman ER, Lepak JM & Angeloni LM (2016) The effect of a single prerelease exposure to conspecific alarm cue on poststocking survival in three strains of rainbow trout (*Oncorhynchus mykiss*). Can J Zool 94: 661–664. dx.doi.org/10.1139/cjz-2016-0086

Kubanek J, Jensen PR, Keifer PA, et al (2003) Seaweed resistance to microbial attack: A targeted chemical defense against marine fungi. Proc Natl Acad Sci U S A 100:6916–6921. <https://doi.org/10.1073/pnas.1131855100>

Kubanek J, Pawlik JR, Eve TM, Fenical W (2000) Triterpene glycosides defend the Caribbean reef sponge Erylus formosus from predatory fishes. Mar Ecol Prog Ser 207:69–77. https://doi.org/10.3354/meps207069

Kynard B & Horgan M (2019) Long‐term studies on restoration of Connecticut River anadromous sea lamprey, *Petromyzon marinus* Linnaeus 1758: Trend in annual adult runs, abundance cycle, and nesting. J Appl Ichthyol 35: 1154–1163. https://doi.org/10.1111/jai.13967

Laframboise AJ, Ren X, Chang S, Dubuc R & Zielinski BS (2007) Olfactory sensory neurons in the sea lamprey display polymorphisms. Neurosci Lett 414(3): 277–281. https://doi.org/10.1016/j.neulet.2006.12.037

Lasne E, Sabatié M, Jeannot N & Cucherousset J (2015) The effects of dam removal on river colonization by sea lamprey *Petromyzon marinus*. River Res Applic 31: 904–911. https:doi.org/10.1002/rra.2789

Lawrence BJ & Smith RJF (1989) Behavioral response of solitary fathead minnows, *Pimephales promelas*, to alarm substance. J Chem Ecol 15(1): 209-219.

Li K, Brant CO, Huertas M, Hessler EJ, Mezei G, Scott AM, Hoye TR & Li W (2018) Fatty-acid derivative acts as a sea lamprey migratory pheromone. PNAS 115(34): 8603–8608. https://doi.org/10.1073/pnas.1803169115

Li W, Scott AP, Siefkes MJ, Yan H, Liu Q, Yun SS & Gage DA (2002) Bile acid secreted by male sea lamprey that acts as a sex pheromone. Science 296(5565): 138–141.

Lima SL & Bednekoff PA (1999) Temporal variation in danger drives antipredator behavior: The predation risk allocation hypothesis. Am Nat 153(6): 649–659.

Lönnstedt OM & McCormick MI (2011) Chemical alarm cues inform prey of predation threat: the importance of ontogeny and concentration in a coral reef fish. Anim Behav 82(2): 213–218. https://doi.org/10.1016/j.anbehav.2011.04.015

Lucon-Xiccato T, Di Mauro G, Bisazza A & Bertolucci C (2020) Alarm cue-mediated response and learning in zebra fish larvae. Behav Brain Res 380: 112446. https://doi.org/10.1016/j.bbr.2019.112446

Luhring TM, Meckley TD, Johnson NS, Siefkes MJ, Hume JB & Wagner CM (2016) A semelparous fish continues upstream migration when exposed to alarm cue, but adjusts movement speed and timing. Anim Behav 121: 41–51. <https://doi.org/10.1016/j.anbehav.2016.08.007>

Mathis A, Smith RJF (1993) Fathead minnows, Pimephales promelas, learn to recognize northern pike, Esox lucius, as predators on the basis of chemical stimuli from minnows in the pike’s diet. Anim Behav 46: 645-656.

Mathuru AS, Kibat C, Cheong WF, Shui G, Wenk MR, Friedrich RW & Jesuthasan S (2012) Chondroitin fragments are odorants that trigger fear behavior in fish. Curr Biol 22(6): 538–544. https://doi.org/10.1016/j.cub.2012.01.061

McCann EL, Johnson NS, Hrodey PJ & Pangle KL (2018) Characterization of sea lamprey stream entry using dual-frequency identification sonar. Trans Am Fish Soc 147(3): 514–524. https://doi.org/10.1002/tafs.10052

Miehls S, Sullivan P, Twohey M, Barber J, & McDonald R (2020) The future of barriers and trapping methods in the sea lamprey (*Petromyzon marinus*) control program in the Laurentian Great Lakes. Rev Fish Biol Fish 30: 1–24. https://doi.org/10.1007/s11160-019-09587-7

Mirza RS & Chivers DP (2001) Are chemical alarm cues conserved within salmonid fishes? J Chem Ecol 27(8): 1641–1655.

Mirza RS, Laraby CA & Marcellus AM (2013) Knowing your behaviour: The importance of behavioural assays in the characterisation of chemical alarm cues in fishes and amphibians. In: East ML & Denhard M (eds) Chemical Signals in Vertebrates, 12^th^ edn. Springer, New York, pp 295-308. https://doi.org/10.1007/978-1-4614-5927-9_24

Mitchell MD, Cowman PF & McCormick MI (2012) Chemical alarm cues are conserved within the coral reef fish Family Pomacentridae. PLoS One 7(10): 1–7. https://doi.org/10.1371/journal.pone.0047428

Moore FR (2018) Biology of landbird migrants: A stopover perspective. Wilson j ornithol 130(1): 1–12.

Parker JD, Collins DO, Kubanek J, et al (2006) Chemical defenses promote persistence of the aquatic plant Micranthemum umbrosum. J Chem Ecol 32:815–833. https://doi.org/10.1007/s10886-006-9038-7

Parra KV, Adrian JC & Gerlai R (2009) The synthetic substance hypoxanthine 3-N-oxide elicits alarm reactions in zebrafish (*Danio rerio*). Behav Brain Res 205: 336–341. https://doi.org/10.1016/j.bbr.2009.06.037

Pereira E, Quintella BR, Mateus CS, Alexandre CM, Belo AF, Telhado A, Quadrado MF & Almeida PR (2017) Performance of a vertical‐slot fish pass for the sea lamprey *Petromyzon marinus* L and habitat recolonization. River Res Applic 33: 16–26. https://doi.org/10.1002/rra.3054

Pfeiffer W, Riegelbauer G, Meier G & Scheibler B (1984) Effect of Hypoxanthine-3(N)-Oxide and Hypoxanthine-1(N)-Oxide on central nervous excitation of the black tetra *Gymnocorymbus ternetzi* (Characidae, Ostariophysi, Pisces) indicated by dorsal light response. J Chem Ecol 11(4): 507–523.

Poisson A, Valotaire C, Borel F, Bertin A, Darmaillacq AS, Dickel L & Colson V (2017) Embryonic exposure to a conspecific alarm cue triggers behavioural plasticity in juvenile rainbow trout. Anim Behav 133: 35–45. https://doi.org/10.1016/j.anbehav.2017.09.013

Robinson TC, Sorensen PW, Bayer JM & Seelye JG (2009) Olfactory sensitivity of pacific lampreys to lamprey bile acids. Trans Am Fish Soc 138(1): 144–152. https://doi.org/10.1577/T07-233.1

Roux O & Diabate A (2014) Divergence in threat sensitivity among aquatic larvae of cryptic mosquito species. J Anim Ecol 83(3): 702–711. https://doi.org/10.1111/1365-2656.12163

Sabal MC, Boyce MS, Charpentier CL, Furey NB, Luhring TM, Martin HW, Melnychuk MC, Srygley RB, Wagner CM, Wirsing AJ, Ydenberg RC & Palkovacs EP (2021) Predation landscapes influence migratory prey ecology and evolution. Trends Ecol Evol 36(8): 737–749. https://doi.org/10.1016/j.tree.2021.04.010

Schoeppner NM & Relyea RA (2009) When should prey respond to consumed heterospecifics? testing hypotheses of perceived risk. Copeia 1: 190–194. https://doi.org/10.1643/CE-08-041

Scott AM, Li K & Li W (2018) The identification of sea lamprey pheromones using bioassay-guided fractionation. J Vis Exp (137): 1–11. <https://doi.org/10.3791/58059>

Scott WB & Crossman EJ (1998). Freshwater Fishes of Canada. Galt House Publications Ltd, Oakville, ON.

Sloychuk JR, Chivers DP, Ferrari MCO (2016) Juvenile lake sturgeon go to school: life-skills training for hatchery fish. Trans Am Fish Soc 145(2): 287-294. https://doi.org/10.1080/00028487.2015.1123183

Smith RJF (1992) Alarm signals in fishes. Rev Fish Biol Fish 2(1): 33–63.

Sorensen PW, Vrieze LA & Fine JM (2004) A multi-component migratory pheromone in the sea lamprey. Fish Physiol Biochem 28(1): 253–257.

Sorensen PW & Vrieze LA (2003) The chemical ecology and potential application of the sea lamprey migratory pheromone. J Great Lakes Res 29: 66–84.

Veen T, Richardson DS, Blaakmeer K & Komdeur J (2000) Experimental evidence for innate predator recognition in the Seychelles warbler. Proc R Soc B: Biol Sci 267(1459): 2253-2258. https://doi.org/10.1098/rspb.2000.1276

Vrieze LA, Bergstedt RA & Sorensen PW (2011) Olfactory-mediated stream-finding behavior of migratory adult sea lamprey (*Petromyzon marinus*). Can J Fish Aquat Sci 68(3): 523–533.

Wagner CM, Bals JD, Hanson ME & Scott AM (2022) Attenuation and recovery of an avoidance response to a chemical anti-predator cue in an invasive fish: implications for use as a repellent in conservation. Cons Phys 10(1): https://doi.org/10.1093/conphys/coac019

Wagner CM, Kierczynski KE, Hume JB & Luhring TM (2016) Exposure to a putative alarm cue reduces downstream drift in larval sea lamprey *Petromyzon marinus* in the laboratory. J Fish Biol 89(3): 1897–1904. https://doi.org/10.1111/jfb.13095

Wagner CM, Stroud EM & Meckley TD (2011). A deathly odor suggests a new sustainable tool for controlling a costly invasive species. Can. J. Fish. Aquat. Sci., 68(7), 1157–1160. https://doi.org/10.1139/F2011-072

Wagner CM, Jones ML, Twohey MB & Sorensen PW (2006) A field test verifies that pheromones can be useful for sea lamprey (*Petromyzon marinus*) control in the Great Lakes. Can J Fish Aquat Sci 63(3): 475–479. https://doi.org/10.1139/F06-008

Wagner CM, Twohey MB & Fine JM (2009) Conspecific cueing in the sea lamprey: do reproductive migrations consistently follow the most intense larval odour? Anim Behav 78(3): 593–599. https://doi.org/ 10.1016/j.anbehav.2009.04.027

William F & Beamish H (1979) Migration and spawning energetics of the anadromous sea lamprey, *Petromyzon marinus*. Environ Biol Fishes 4(1): 3–7.

Wisenden BD (2000) Olfactory assessment of predation risk in the aquatic environment. Philos Trans R Soc B Biol Sci 355(1401): 1205–1208. <https://doi.org/10.1098/rstb.2000.0668>

Wisenden BD (2015) Chemical cues that indicate risk of predation. In: Sorensen PW, Wisenden BD (ed) Fish pheromones and related cues. Wiley- Blackwell Press, Hoboken, New Jersey, pp. 131–148.

Wisenden BD, Vollbrecht KA & Brown JL (2004) Is there a fish alarm cue? Affirming evidence from a wild study. Anim Behav 67(1): 59-67. https://doi.org/10.1016/j.anbehav.2003.02.010

Yambe H, Kitamura S, Kamio M, Yamada M, Matsunaga S & Fusetani N (2006) L-Kynurenine, an amino acid identified as a sex pheromone in the urine of ovulated female masu salmon. PNAS 103(42): 15370–15374. https://doi.org/10.1073/pnas.0604340103

Zielinski B, Arbuckle W, Belanger A, Corkum LD, Li W & Scott AP (2004) Evidence for the release of sex pheromones by male round gobies (*Neogobius melanstomus*). Fish Physiol Biochem 28: 237–239

STATEMENTS AND DECLARATIONS

**Funding**

This work was supported by the Great Lakes Fishery Commission.

**Competing Interests**

The authors state that they have no conflict of interests to declare.

**Author Contributions**

All authors contributed to the study conception and design. Amila A. Dissanayake and Muraleedharan G. Nair chemically isolated and identified compounds. Emily L. Mensch and C. Michael Wagner conducted behavioral experiments and analyzed data. The first draft of the manuscript was written by Emily L. Mensch and C. Michael Wagner, and all authors commented on previous versions of the manuscript. All authors read, revised, and approved the final manuscript.


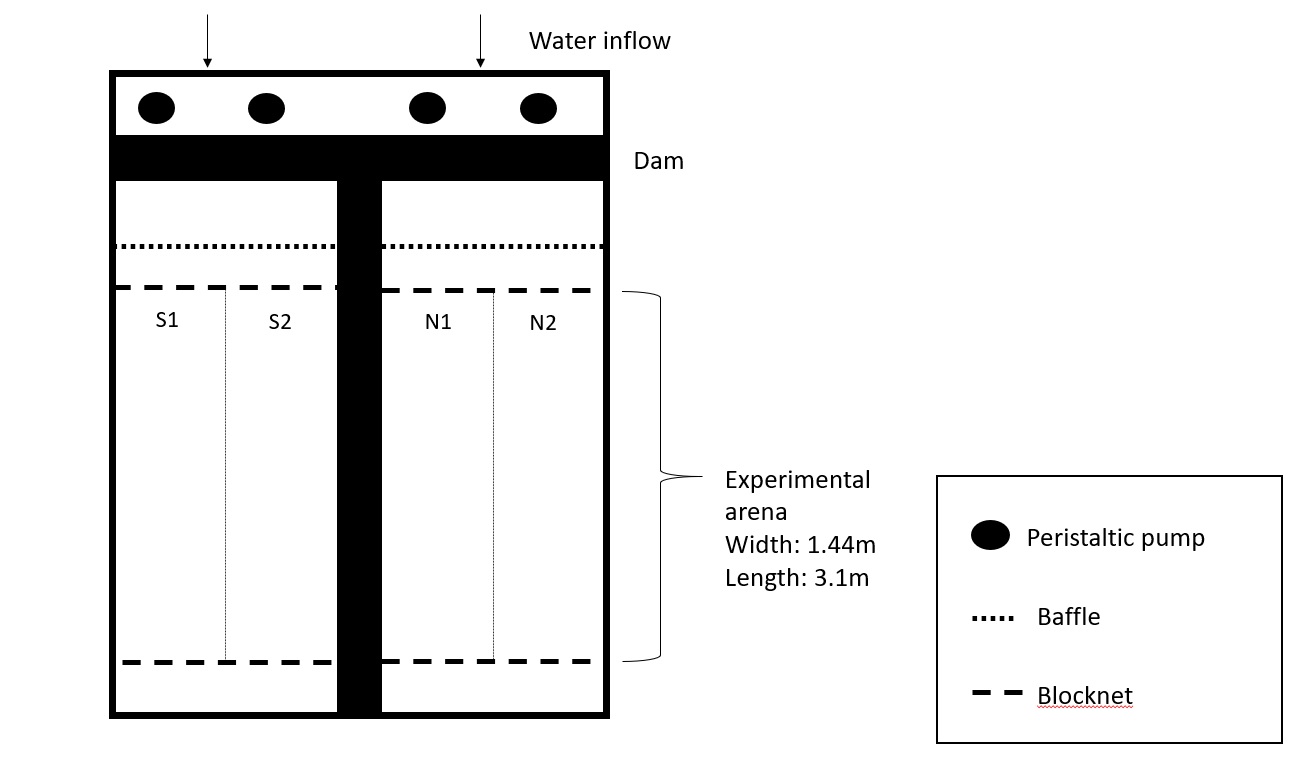


**Fig. 1** Schematic of laboratory raceway. Fish were introduced into middle of either south (S1 and S2) or north (N1 and N2) raceways at beginning of trial. Odor was introduced via one peristaltic pump during the "stimulus" period of the trial, and pump sides were switched between each trial to account for side bias


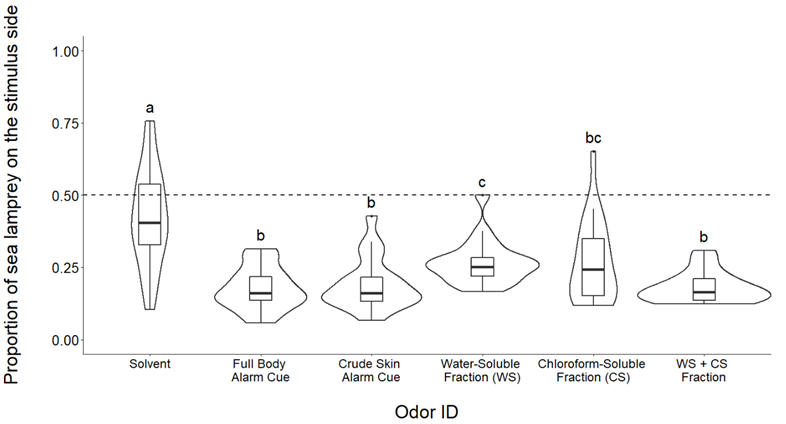


**Fig. 2** Boxplots representing the proportion of sea lamprey on the stimulus side after the addition of odorants. The middle quartile of boxes represents treatment median, and upper and lower quartiles are the 75^th^ and 25^th^ percentile of the range, respectively. Upper and lower whiskers represent the minimum and maximum spread of the data. Violin plots demonstrate the frequency of proportion values for each treatment. WS = water-soluble and CS = chloroform soluble. Dashed line at 0.50 represents the null hypothesis of a true neutral response to introduced stimulus. Treatments with different letters are significantly different from one another based on Tukey HSD (α = 0.05). N = 20 for each bar

*
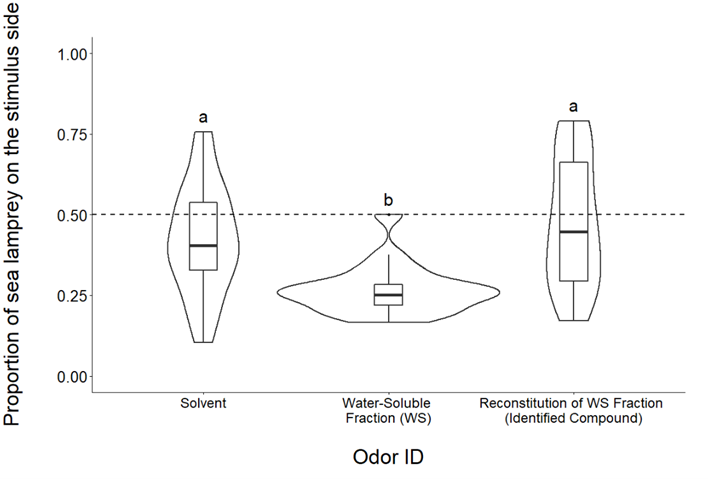
*

**Fig. 3** Boxplots representing the proportion of sea lamprey on the stimulus side after the addition of odorants. The middle quartile of boxes represent treatment median, and upper and lower quartiles are the 75^th^ and 25^th^ percentile of the range, respectively. Upper and lower whiskers represent the minimum and maximum spread of the data. Violin plots demonstrate the frequency of proportion values for each treatment. Dashed line is null hypothesis of 50% of animals on the stimulus side after the introduction of the odor. Treatments with different letters are significantly different from one another based on Tukey HSD (α = 0.05). N = 20 for each bar

**Table 1** List of individual compounds and subfractions tested in screenings of individual compounds and subfractions. P-values are derived from one-way two-sided T-tests comparing proportion of sea lamprey on the stimulus side after introduction of odorant to a null-hypothesis of 50:50 proportion of sea lamprey on the stimulus side (*p< 0.05; **p< 0.01; ***p< 0.001).

| Treatment | P-value | Mean | N |
| --- | --- | --- | --- |
| Solvent | 0.725 | 0.517 | 20 |
| Whole Body Alarm Cue | 3.6e-07 *** | 0.323 | 37 |
| Arginine | 0.417 | 0.408 | 5 |
| Valine | 0.731 | 0.537 | 5 |
| Isoleucine | 0.036 * | 0.371 | 10 |
| Leucine | 0.456 | 0.582 | 5 |
| Hypoxanthine | 0.062 | 0.381 | 10 |
| Tyrosine | 0.222 | 0.441 | 10 |
| Phenylalanine | 0.785 | 0.431 | 5 |
| Inosine | 0.121 | 0.582 | 5 |
| Tryptophan | 0.090 | 0.651 | 5 |
| Glutamic acid | 0.374 | 0.610 | 5 |
| Histidine | 0.528 | 0.465 | 5 |
| Creatine | 0.933 | 0.483 | 5 |
| Isoleucine + Tyrosine + Hypoxanthine | 0.886 | 0.487 | 5 |
| Pure Compound 1 (Petromyzonicil) | 0.700 | 0.46 | 5 |
| Chondroitin Sulfate | 0.351 | 0.578 | 5 |
| Subfraction SL-3 (Creatine + Arginine) | 0.336 | 0.546 | 10 |
| Subfraction SL-4 (Creatine + Arginine + Valine + Leucine + Isoleucine) | 0.034* | 0.319 | 5 |
| Subfraction SL-5 (Hypoxanthine + Inosine) | 0.333 | 0.439 | 10 |
| Subfraction SL-6 (Adenine + Tyrosine + Xanthine) | 0.869 | 0.491 | 10 |
| Subfraction SL-7 (Histidine + Phenylalanine + Glutamic Acid + Tryptophan + Threonine) | 0.860 | 0.514 | 10 |
| Subfraction SL-8 (Asparagine + Methionine + Cysteine + Adenosine + Glycine) | 0.430 | 0.566 | 5 |

**Table 2** List of the identified compounds in the water-soluble fraction and the percent of the whole fraction each compound constitutes. The water-soluble fraction is 48.70% of the entire crude skin extract and the chloroform-soluble fraction is 51.30%.

| Compound | % | Compound | % | Compound | % | Compound | % |
| --- | --- | --- | --- | --- | --- | --- | --- |
| Creatine | 36.95% | Histidine | 0.18% | Serine | 0.02% | Putrescine | 0.04% |
| Arginine | 1.92% | Tryptophan | 0.53% | Aspartic acid | 0.19% | Spermine | 0.02% |
| Valine | 0.24% | Threonine | 1.25% | Inosine | 0.22% | 3-Phenyllactic acid | 0.10% |
| Leucine | 0.51% | Asparagine | 1.17% | Adenine | 0.17% | Pyruvic acid | 0.03% |
| Tyrosine | 0.08% | Methionine | 0.41% | Xanthine | 0.36% | β-Hydroxybutyric acid | 0.06% |
| Isoleucine | 0.17% | Glycine | 0.24% | Hypoxanthine | 0.85% | α-Ketobutyric acid | 0.07% |
| Phenylalanine | 1.29% | Cysteine | 0.84% | Adenosine | 0.48% | α-Ketoisovaleric acid | 0.06% |
| Glutamic acid | 0.09% | Proline | 0.07% | Petromyzonacil | 0.04% | α-Ketovaleric acid | 0.01% |
